# Application of RNA-seq for mitogenome reconstruction, and reconsideration of long-branch artifacts in Hemiptera phylogeny

**DOI:** 10.1038/srep33465

**Published:** 2016-09-16

**Authors:** Nan Song, Shiheng An, Xinming Yin, Wanzhi Cai, Hu Li

**Affiliations:** 1 College of Plant Protection, Henan Agricultural University, Zhengzhou, China; 2 Department of Entomology, China Agricultural University, Beijing, China

## Abstract

Hemiptera make up the largest nonholometabolan insect assemblage. Despite previous efforts to elucidate phylogeny within this group, relationships among the major sub-lineages remain uncertain. In particular, mitochondrial genome (mitogenome) data are still sparse for many important hemipteran insect groups. Recent mitogenomic analyses of Hemiptera have usually included no more than 50 species, with conflicting hypotheses presented. Here, we determined the nearly complete nucleotide sequence of the mitogenome for the aphid species of *Rhopalosiphum padi* using RNA-seq plus gap filling. The 15,205 bp mitogenome included all mitochondrial genes except for *trnF*. The mitogenome organization and size for *R. padi* are similar to previously reported aphid species. In addition, the phylogenetic relationships for Hemiptera were examined using a mitogenomic dataset which included sequences from 103 ingroup species and 19 outgroup species. Our results showed that the seven species representing the Aleyrodidae exhibit extremely long branches, and always cluster with long-branched outgroups. This lead to the failure of recovering a monophyletic Hemiptera in most analyses. The data treatment of Degen-coding for protein-coding genes and the site-heterogeneous CAT model show improved suppression of the long-branch effect. Under these conditions, the Sternorrhyncha was often recovered as the most basal clade in Hemiptera.

Hemiptera is the largest nonholometaboan group of insects, with approximately 82,000 described species^1^. The hemipteran insects have distinctive piercing-and-sucking mouthparts, which make them better adapt to extensive evolutionary radiation^2^. Many species are the important insect pests due to their high reproductive rates and characteristic ability of transmitting human and plant diseases. Despite the significant importance of Hemiptera in biology, the phylogenetic relationships within this group remain unresolved. In particular on the deep-level relationships, almost every possible arrangement among super-families has been proposed. Different relationships of super-families lead to incongruent hypotheses on inter-suborder relationships. Traditionally, two insect groupings rank as suborders or orders: Homoptera and Heteroptera, constituting the Hemiptera^3^,^4^,^5^. The former includes the two suborders Sternorrhyncha and Auchenorrhyncha, while the latter comprises the Heteroptera and Coleorrhyncha (which together form clade Heteropterodea)^6^. However, numerous morphological and molecular studies have shown that Homoptera is not a monophyletic group^7^,^8^,^9^,^10^,^11^ (e.g., Fig. 1A–C). More recently, mitogenomic^12^,^13^ and whole genomic^14^ data also recovered the Sternorrhyncha as the sister group to all other Hemiptera, rendering the Homoptera to be a paraphyletic group (e.g., Fig. 1D). In contrast, some other researches supported the monophyly of Homoptera^15^,^16^,^17^, that is the Cicadomorpha, Fulgoromorpha and Sternorrhyncha clustered in one clade (e.g., Fig. 1E,F). The key to determine whether Homoptera is monophyletic or not is in the placement of Sternorrhyncha. Besides the question on the Homptera, the monophyly of Auchenorrhyncha^18^ and the position of Coleorrhyncha^14^,^17^,^19^ are also the focus of the debate on the higher-level phylogeny of Hemiptera.

The debate may be due, in part, to the unbalanced distribution of mitogenomic studies among suborders of Hemiptera. Traditionally, the order Hemiptera are comprised of three suborders: Sternorrhyncha (aphids, psyllids, whiteflies, and coccids), Auchenorrhyncha (cicadas, spittlebugs, leafhoppers, treehoppers, and planthoppers), and Heteroptera (true bugs)^20^,^21^. To date, the determined complete or nearly complete mitogenomes are mainly concentrated in the suborder Heteroptea (61 sequenced heteroptean mitogenomes, data to January 2015), while limited mitogenomes from suborder Sternorrhyncha can be available in GenBank (only 19 sternorrhynchan species of total 103 hemipterans). Therefore, increasing the number of taxa sampled, particularly in Sternorrhyncha, is needed to robustly test the value of mitogenome data in resolving relationships within Hemiptera. Classically, most insect mitogenomes were sequenced by long PCR plus primer walking under a series of designing conserved PCR primers. However, this method is time consuming and inefficient due to the varied amplification conditions to different insect lineages. In comparison, retrieving mitogenome data from transcriptome sequencing could overcome the difficulty of long PCR and cumbersome primer designs, which typically includes all of the mitochondrial protein-coding and rRNA genes^22^. The thirteen mitochondrial protein-coding genes (PCGs) and two rRNA genes contain the vast majority of phylogenetic information of the whole mitogeome^23^, and can meet the requirement of inferring phylogeny using majority of mitogenome data.

*Rhopalosiphum padi* (Aphididae) is an economically important pest on wheat and the main vector of barley yellow dwarf virus on cereals in the world. Infection with barley yellow dwarf virus causes wheat to turn yellow, and further leads to grain number and weight to reduce sharply. Affected plants are generally severely stunted and non-productive. The resulting losses in grain yield are often more than 40% in China^24^. Although *R. padi* has been the subject of extensive biological and molecular studies, phylogenetic study of this insect species is limited. According to prior studies on Hemiptera phylogeny^12^,^15^, the clade of aphids have relatively lower evolutionary rate than whiteflies in the suborder Sternorrhyncha, and the former usually display shorter branch length. Additional mitogenome sequence from aphids, for example the *R. padi*, can be helpful to ameliorate the long-branch problems in Hemiptera.

In the present study, we applied the method of RNA-seq plus gap filling to *R. padi* mitogenome determination. The nearly complete mitogenome of *R. padi* was sequenced and annotated, with the exception of the *trn*F locus, which required direct sequencing. In addition, improved phylogenetic analyses with increased mitogenome data (including one newly sequenced mitogenome of *R. padi* for this study plus 121 published insect mitogenomes from GenBank) were utilized to investigate the Hemiptera relationships, with an emphasis on the placement of Sternorrhyncha. To reduce the effect of sequence saturation and compositional heterogeneity, which are potentially related to long-branch attraction artifact, the most comprehensive methods of Hemiptera phylogeny including a series of data coding schemes, locus refinement, phylogenetic inference method, and model settings are performed for tree reconstruction.

## Methods

### Sampling strategy

Lab-reared populations of *R. padi* used for high-throughput sequencing and mitogenome assembly were originally collected in China (Zhengzhou, Henan province, November 2014). Total RNA was extracted from 35 to 50 aphid individuals using Trizol reagent (Invitrogen, CA, USA) following the manufacturer’s protocol.

### Construction of *R. padi* mitogenome

Total RNA was quantified using a NanoPhotometer spectrophotometer (IMPLEN, CA, USA) and RNA quality was verified using a 2100 Bioanalyzer with the RNA Nano 6000 Assay Kit (Agilent Technologies, CA, USA). The cDNA library were constructed using IlluminaTruSeq RNA Sample Preparation Kit (Illumina, San Diego, CA, USA). The sequence sample contained >50 μg of total RNA, which was diluted with nuclease-free ultrapure water to a final volume of 50 μl before mRNA was purified from total RNA using poly-T oligo-attached magnetic beads. The purified mRNA was fragmented and used for first strand cDNA synthesis using reverse transcriptase Super Script II and random hexamers. The RNA templates were then removed using RNaseH and the second strand was synthesized using DNA polymerase I to generate double-stranded cDNA fragments. The purified cDNA fragments were repaired on the 3′ end and adenylated before being ligated to sequencing adapters. Size selection was performed using AMPure XP beads. Finally, the cDNA fragments were enriched by PCR amplification using random hexamers, and products were purified by AMPure XP beads to generate final library. The size and purity of the cDNA sequencing libraries were determined using a 2100 Bioanalyzer with the RNA Nano 6000 Assay Kit (Agilent Technologies, CA, USA) and the quantity was estimated using Q-PCR. RNA transcript was sequenced on an Illumina Hiseq 2000 in Novogene Bioinformatics Institute (Beijing, China). In total, 62,468,816 raw data of paired-end reads of 100 bp length were generated in a lane.

Raw data of fastq format were processed through in-house perl scripts. In this step, reads containing adapters, reads containing ploy-N, and low quality reads were removed from raw data. At the same time, Q20, Q30, GC-content and sequence duplication level of the cleaned data were calculated. All the downstream analyses were based on clean data with high quality. A global *de novo* assembly of the resultant reads was performed using the Trinity method with min_kmer_cov set to 2 by default and all other parameters set default. To annotate the obtained unigenes, the databases of NR (with a cutoff evalue ≤1e-5), NT (evalue ≤1e-5), KO (evalue ≤1e-5), Swiss-Prot (evalue ≤1e-5), PFAM (evalue ≤1e-5), GO (evalue ≤1e-6), and KOG/COG (evalue ≤1e-3) were searched. The resultant data were inputted into BioEdit version 7.0.5.3^25^ to build a local BLAST to search mitochondrial genes, with the published aphid mitogenomes (mainly using *Schizaphis graminum* and *Sitobion avenae*)^26^,^27^ as bait sequences.

Through local BLAST searching, twelve complete or partial sequences of mitochondrial PCGs were found in the RNA-seq results, while the *atp8* was missing. In addition, partial regions of two mitochondrial rRNA genes (1200 bp 3′ end of *rrnL* and 322 bp 5′ end of *rrnS*) and the full length *trnV* gene were identified in the transcriptome assembly. The remaining mitochondrial gene fragments were sequenced from genomic DNA by designing primers and PCR amplification (primers are listed in Table S1). Total genomic DNA was isolated using Qiagen DNA extraction kits (Qiagen, Beijing, China). The species-specific primers were designed based on the sequences from the RNA-seq, and by referring to Simon *et al.*^28^. The PCR cycling parameters were as follows: initial denaturation of 5 min at 94 °C; 35 cycles of 30 s at 95 °C, 30 s at the annealing temperature 45–55 °C, 1–2 min elongation at 72 °C; and a final elongation of 10 min at 72 °C. The PCR products were electrophoretically inspected in 1.5% agarose gels, and directly sequenced after purification. DNA sequencing was performed with a BigDye Terminator Cycle Sequencing Kit and an ABI 3730XL Genetic Analyzer (PE Applied Biosystems, USA).

The mitochondrial DNA sequences were assembled by SeqMan as implemented in Lasergene software package (DNAStar, Inc.). The finally assembled mitogenome sequences were annotated by MITOS^29^ on the Invertebrate Genetic Code, and other published aphid mitogenomes^26^,^27^,^30^,^31^. The image of *R. padi* mtiogenome organization was generated with mtviz http://pacosy.informatik.uni-leipzig.de/mtviz/ (Fig. 2). New mitogenome sequence obtained in this study was deposited in GenBank under accession number of KT447631.

### Phylogenetic reconstruction and tests

Table S2 lists the species included in the phylogenetic analyses, the taxon status and the GenBank accession numbers. Totally 103 mitogenomes from Hemiptera comprised ingroup and 19 from Orthoptera, Psocoptera, Thysanoptera, and Phthiraptera were selected as outg. Each of the 37 mitochondrial genes were aligned for further analyses. For PCGs stop codons were excluded, each was aligned separately based on the invertebrate mitochondrial genetic code with Perl script transAlign^32^, and then each alignment was concatenated in a single matrix using FASconCAT_v1.0^33^. Both the mitochondrial tRNA and rRNA genes were aligned with reference to the conserved secondary structure. Every tRNA gene was aligned manually: first, the anticodon was located for each gene; second, the seven base-paired anticodon arm sequences were found; third, separately from both sides of the sequence, the acceptor arm sequences were partitioned; finally, the highly variable regions (DHUarm, TψC arm and variable loop of them) were refined by MUSCLE in MEGA version 6.0^34^. All 22 tRNA gene alignments were concatenated by FASconCAT to construct the tRNAs dataset. Each of the two rRNAs was aligned separately by the R-Coffee web server^35^, and the aligned sequences were refined by eye and compiled as the dataset of rRNAs.

Nucleotide composition of these sequences including the *R. padi* determined in this study was calculated using MEGA. GC-skew values were calculated under the formula: (G–C)/(G + C). Sequence potential saturation was assessed using the index of substitution saturation (*Iss*) of Xia *et al.*^36^ implemented in DAMBE5^37^. To detect nucleotide homogeneity across taxa, the chi-square test was performed for the concatenated datasets using PAUP 4.0b10^38^. Estimates of synonymous (*dS*) and nonsynonymous (*dN*) substitution rates of each PCG were obtained using the program yn00 of PAML package^39^. And the Orthoptera was used as references. Based on the results from substitution rate analysis, the relatively conservative gene locus were selected to construct the matrix to reduce the effect of rapid evolutionary rate of mtDNA on tree building. That is the dataset of 122taxa_PCG_7genes_AA: the amino acid sequences of 122 taxa from seven PCGs (i.e., *atp6*, *cox1*, *cox2*, *cox3*, *cytb*, *nad1* and *nad4*).

To eliminate the effect of saturation and compositional heterogeneity on phylogenetic reconstruction, we applied six data coding strategies to the sequences of PCGs: 1) nucleotide sequences with all codon positions (PCG123); 2) nucleotide sequences removing third codon positions (PCG12); 3) RY-coding the third codon positions (PCG3RY); 4) the first codon positions RY-coded plus the second codon positions (PCG1RY2nd); 5) including only nonsynonymous changes at all coding positions through Degen v1.4^40^,^41^ (PCGDegen); 6) the deduced amino acid matrix (PCG_AA).

To test effect of combined analysis on the tree building, sequences of RNA genes were concatenated into the PCGs alignments. Four correspoding datasets were compiled: 1) PCGRNA: 13 PCGs, two rRNAs and 22 tRNAs with 14,502 nucleotides; 2) PCG12RNA: 13 PCGs removing the third codon positions, two rRNAs and 22 tRNAs with 10,819 nucleotides; 3) PCG1RY2ndRNA: 13 PCGs with 1RY+2nd coding strategy, two rRNAs and 22 tRNAs with 10,819 nucleotides; 4) PCGDegenRNA: 13 PCGs with Degen-coding strategy, two rRNAs and 22 tRNAs with 14,502 nucleotide. In addition, to investigate the effect of taxon sampling on tree topology, 106 taxa dataset with all genes (i.e., 106taxa_PCGRNA) were created.

Tree searches were conducted on each of three types of genes (PCGs, tRNAs and rRNAs) and on the combined dataset. In total, fourteen datasets were analyzed using both Maximum likelihood (ML) and Bayesian inference (BI) (Table 1). Before undertaking ML analyses, PartitionFinder^42^ was employed to infer the optimal partitioning strategy, meanwhile the best-fitting model was selected for each partition using the Bayesian Information Criterion (BIC). The data blocks were defined by gene types (each of 13 PCGs, 22 tRNAs and 2 rRNAs separately as independent block) and by codon positions. The partition schemes and best-fitting models were calculated for 122taxa_PCGRNA and 122taxa_PCG_AA, and 106taxa_PCGRNA, respectively (Table S3). ML searches were carried out using the partition schemes and the selected models described above with RAxML as implemented in the CIPRES Portal^43^. Support for nodes was assessed with the fast bootstrap method using 1000 non-parametric bootstrap inferences.

The BI analyses were initially conducted using MrBayes_v.3.2^43^,^44^ with the following priors: independent substitution model for each partition separated by genes and codons, all model parameters were unlinked across partitions, four Markov chains, two independent runs each for 10 million generations, sampling every 1000th generation, and the first 25% discarded as burn-in. Convergence was considered to be reached when the average standard deviation of split frequencies fell below 0.01. BI analyses were also performed by PhyloBayes with a parallel version (pb_mpi1.5a)^45^,^46^ as implemented on a HP server with twenty-four CPU and 64 G memory. The model GTR-CAT was used for nucleotide analyses, while the model CAT for amino acids. Two chains were run, and started from a random topology. The Maximum\maxdiff” value to accept was set as 0.1. All sequence alignment files and original tree files constructed in this article are available in the TreeBASE http://purl.org/phylo/treebase/phylows/study/TB2:S18206.

**Figure 1:**
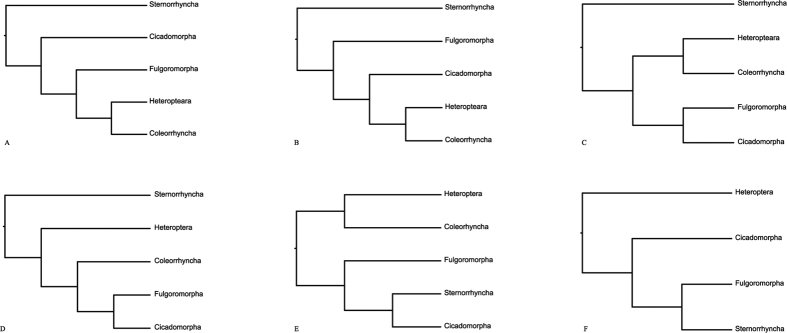
Alternative hypotheses of deep-level relationships within Hemiptera: (**A)** Campbell *et al.*^9^; (**B)** Bourgoin & Campbell^8^ paraphyletic Homoptera due to the most basal position of Sternorrhyncha based on 18SrDNA sequences; (**C)** Cryan & Urban^18^ paraphyletic Homoptera due to the most basal position of Sternorrhyncha based on nuclear and mitochondrial gene sequences; (**D)** Misof *et al.*^14^ paraphyletic Homoptera due to the most basal position of Sternorrhyncha based on genome-scale data; (**E)** Hamilton^17^ monophyletic Homoptera as sister group to Heteropterodea based on morphological characters; and (**F)** Song *et al.*^15^ monophyletic Homoptera based on limited mitogenomic data.

To test statistically the conflict between alternative hypotheses of Hemiptera phylogeny, we compared relations proposed in previous studies (e.g., the basal position of Sternorrhyncha in Fig. 1A: Campbell *et al.*^9^ and Fig. 1D: Misof *et al.*^14^; the intermediate position of Sternorrhyncha in Fig. 1F: Song *et al.*^15^) to the molecular phylogeny obtained in this study. The Shimodaira-Hasegawa test (SH)^47^ and the approximately unbiased test (AU)^48^ were conducted for a series of datasets with 122 taxa. The site-log-likelihood values were calculated under the GTR + I +G model for nucleotides and the MtREV + I + G model for amino acids using TREE-PUZZLE 5.3^49^. The obtained values were used as input for the software CONSEL^50^. Constraint likelihood trees were constructed on the basis of dataset of 122taxa_PCGRNA using RAxML as implemented above.

## Results

### *R. padi* Mitogenome

After removing the adaptors and filtering the low quality reads, Illumina sequencing produced a total number of 30,582,489 of clean reads for each end, and the percentage of clean reads were about 98% for Q20, and 93% for Q30. The total error rate of both end sequencing was 0.03%, indicating a high quality of sequence recovered. The average GC contents were 40.15% and 40.22% for each sequencing. Based on the clean reads, a total of 36,888 contigs (≥200 bp) without gaps was generated by Trinity. Contigs size ranged from 201–17,707 bp, with an average length of 1,359 bp and N50 of 2,416 bp, respectively. Of the 24,782 unigenes, 12,702 unigenes (51.3%) ranged from 200–500 bp, and 3770 unigenes (15.3%) were >2000 bp.

**Figure 2:**
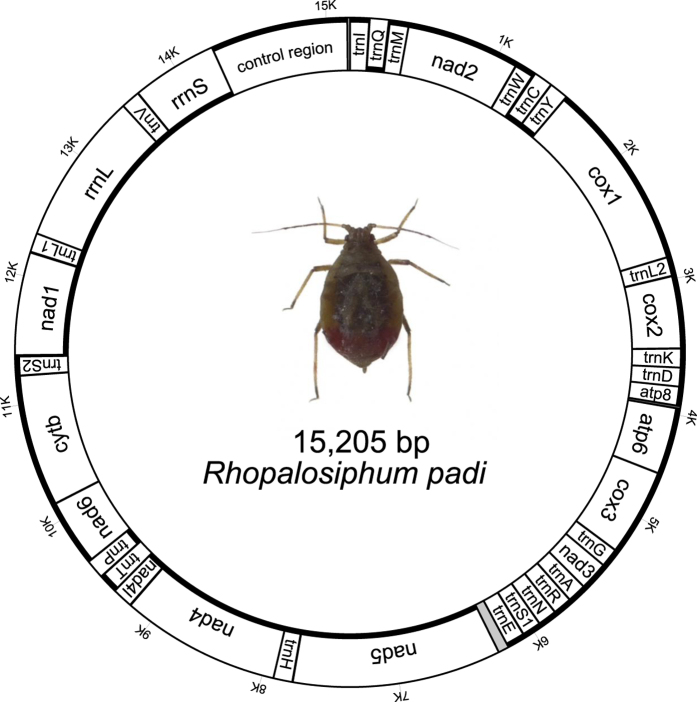
Graphical representation of the mitochondrial genome of *Rhopalosiphum padi*. The coding strand is indicated by think line; and abbreviations are as in the text.

After RNA-seq and directly sequencing for gap filling, the final assembly of mitogenome of *R. padi* contained 13 PCG, 2 rRNA and 21 tRNA genes, and a putative control region (Fig. 2, and Table 2). The only gap region not being determined was located between *trnE* and *nad5*. Though we successfully amplified this region by PCR reactions, yet sequencing consistently failed due to higher A + T content or highly repetitive sequences within it. The mitogenome of *R. padi* was very compact, twelve overlaps (a total of 42 bp) between adjacent genes were observed. The intergenic spacers were relatively small, except for the gap region found between *trnE* and *nad5*. The largest one (10 bp) was found in the region between *trnS*(*UCN*) and *nad1*. All thirteen PCGs in *R. padi* started with the typical codon ATN. Specifically, two genes (*cox3* amd *cytb*) started with ATG, three (*atp8*, *atp6* and *nad3*) with ATT, and the remainder with ATA. Besides canonical stop codons (TAA or TAG), a single T was used for *cox1*, *nad4* and *nad5*.

We compared similarity between the mitogenome PCG obtained for *R. padi* with those from the aphid *S. graminum* (from GenBank). Similarities were *atp6* = 91%, *cox1* = *92*%, *cox2* = 93%, *cox3* = 92%, *cytb* = 93%, *nad1* = 94%, *nad2* = 94%, *nad3* = 91%, *nad4* = 95%, *nad4l* = 97%, *nad5* = 95%, and *nad6* = 91%. With regard to another closely related aphid, *S. avenae*, homology between each PCG from two species was similar to that between *R. padi* and *S. graminum* (i.e., *atp6* 89%, *cox1* 92%, *cox2* 92%, *cox3* 88%, *cytb* 89%, *nad1* 93%, *nad2* 88%, *nad3* no hits found, *nad4* 93%, *nad4l* 95%, *nad5* 93%, and *nad6* 92%). For *atp8*, there were no matching sequences identified *R. padi* and *S. graminum* or *R. padi* and *S. avenae*, nor from other aphid mitogenomes.

**Figure 3:**
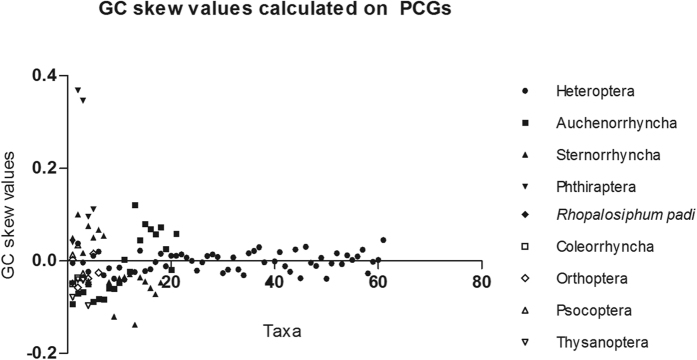
GC-skew values were calculated on dataset of mitochondrial PCGs with all codon positions for *Rhopalosiphum padi*.

The mitogenome of *R. padi* displayed significant bias A + T content, with 82.9% A + T nucleotide composition. GC skew values can be used as a measure for base compositional differences. The GC skews were calculated for PCGs, tRNA and rRNA genes for all taxa included in this study. The results showed that the majority of species representing Sternorrhyncha and outgrups Phthiraptera and Thysanoptera had similarly higher GC skew values over PCGs, tRNAs and rRNAs (Fig. 3 and Fig. S1). Shared nucleotide compositional biases lead to increased homoplasy at unconstrained sites and create erroneous signal^51^, which were suspected to cause long-branch attraction problem.

The standard 21 tRNA genes were identified in the mitogeome of *R. padi*, which ranged from 62 bp [*trnD, trnS(AGN), trnT* and *trnV*] to 73 bp (*trnK*) in size. The two rRNA genes (*rrnL* and *rrnS*) in the *R. padi* mitogenome were located between *trnL(CUN)* and *trnV*, and between *trnV* and the putative control region, respectively (Fig. 2, and Table 2). The lengths of the *rrnL* and *rrnS* genes were respectively 1,259 bp and 767 bp, with the A + T contents of 85.1% and 84.0%. A putative control region was determined between *rrnS* and *trnI*, with length of 745 bp and A + T content of 87.1%. In the insect mitogeome, the control region is also called AT-rich region, like that in *R. padi* with a control region having the highest A + T content through the whole majority strand. Three repeat motifs with about 100 bp elements were detected in the 5′ end of the control region of *R. padi*, which was followed by a [TA(A)]n-like region with 230 bp in size. The 3′ end was composed of a region with higher G + C content.

### Saturation test and evolutionary rate estimate

None of the DAMBE tests yielded an observed index of substitution saturation (*Iss*) greater than the critical value (*Iss.c*), with the exception of the third codon position when NumOTU was 32 (Table 3). For the index of *Iss.cAsym*, only values of *Iss* based on PCG12 and PCG2 were significantly lower than *Iss.cAsym* when NumOTU was 32. The PCG3 failed to pass this test whether NumOTU was 16 or 32. This result suggested that the positions of third codon experienced so much saturation that they had poor phylogenetic information for tree reconstruction. Chi-square tests indicated significant heterogeneity of base composition between taxa in each codon, and each types of gene dataset (*p *< 0.05). Estimates of substitution rates showed that data matrix compiled from *cox1 (dN* = 0.1623), *cytb (dN* = 0.2428), *cox2 (dN* = 0.2803), *cox3 (dN* = 0.3084), *nad1 (dN* = 0.3235), *atp6 (dN* = 0.3475) and *nad4 (dN* = 0.3820) contained fewer non-synonymous substitution sites.

### Phylogenetic analyses

For each tree reconstructed in the phylogenetic analyses, nodal support values for major lineages and corresponding branch lengths are provided in Table 1. In all analyses, a monophyletic Aphidoidea was recovered. In addition, the monophyly of Aphididae and a sister group relationship between *R. padi* and *S. graminum* were supported by the trees from various datasets. These resulting phylogenies confirmed the validity of mitogenome data of *R. padi* sequenced in this study.

### Separate analysis

The mitochondrial rRNA, tRNA and PCG genes represent three different kinds of makers, which resulted in different phylogenetic relationships within Hemiptera. In the ML analyses based on rRNAs dataset, Hemiptera was monophyletic, and the most basal position of Sternorrhyncha was supported, which led to a paraphyletic Homoptera. Although rRNAs MrBayes recovered a monophyletic Hemiptera, the Sternorrhyncha was recovered in a more derived position, and the Homoptera was retained. PhyloBayes analyses under site-heterogeneous model CAT produced a similar tree structure to MrBayes, the only difference was on the placement of Coleorrhyncha.

In contrast, the tRNAs dataset displayed weaker resolution of Hemiptera phylogeny. All tRNAs analyses failed to recover the monophyly of Hemiptera, due to the nested position of Thysanoptera and Phthiraptera.

For PCGs, different data coding strategies (i.e., PCG123, PCG12, PCG3RY, PCG1RY2nd, PCGDegen and PCG_AA) had dramatic effects on the estimated phylogenetic trees. With the site-homogeneous model, ML and MrBayes analyses yielded nearly identical tree topology, where outgroups Thysanoptera and Phthiraptera were always embedded into the ingroup and had a close affinity to the long-branched Sternorrhyncha. Under the heterogeneous model, the PhyloBayes analyses produced better-resolved trees. In the PhyloBayes trees based on the PCG1RY2nd and PCGDegen datasets, the long branches were separated, and outgroups Thysanoptera and Phthiraptera were pulled into a more basal position. By removing the Thysanoptera, the monophyly of Hemiptera were supported by both datasets. Nonetheless, only the PCGDegen recovered a basal placement of Sternorrhyncha thus causing the Homoptera to be a paraphyletic group. The remaining four PCG datasets (i.e., PCG123, PCG3RY, PCG12 and PCG_AA) gave poor results in respect of breaking long-branched assemblages. Though the PhyloBayes analysis based on amino acid sequence from seven relatively conserved PCGs (i.e., the dataset of 122taxa_PCG_7genes_AA) could not recover a monophyletic Hemiptera, the long-branched outgroups were set apart from the ingroup taxa with the longest branches and pulled toward the base of tree. This demonstrated that employing relatively conserved gene regions can reduce long-branch attraction to some extent.

### Combined analysis

Despite with potential saturation, the third codon positions can still contain phylogenetic signal^52^,^53^,^54^. Thus, the third codon positions were included in the combined analyses. ML and MrBayes analyses with combined datasets revealed very similar tree topologies to those from separate analysis. In all these trees, intermediate branching position of Sternorrhyncha was retrieved, and it consistently clustered with Thysanoptera and Phthiraptera.

**Figure 4:**
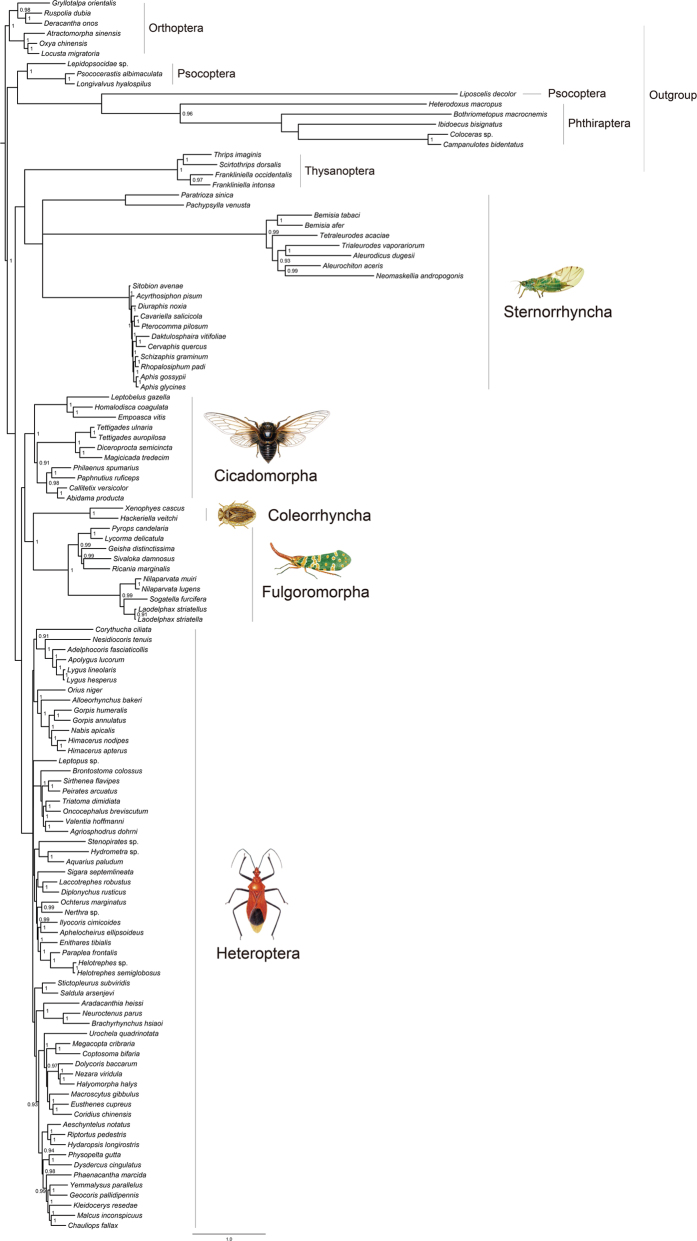
Bayesian tree estimated from dataset of 122taxa_PCGRNA under CATGTR model. The site-heterogeneous model showed significant improvement in suppressing long-branch attraction, thus this topology is presented as one of the most likely tree structures for deep-level phylogeny of Hemiptera. Node numbers show posterior probabilities (above 0.9), and scale bar represents substitutions/site.

PhyloBayes analyses of the combined data using the site-heterogeneous model showed improvement in suppressing long-branch attraction. Four combined PhyloBayes analyses only differed in the extent of breaking long branches. Both PCGRNA and PCG1RY2ndRNA recovered outgroups Thysanoptera and Phthiraptera as the basal clades in the trees. And in both trees, the Sternorrhyncha formed the most basal hemipteran lineage, and was a sister group of all other Hemiptera (Fig. 4). The PCG12RNA and PCGDegenRNA resulted in the topology that only Thysanoptera were recovered outside ingroup. Despite being separated away from the longest branching Sternorrhyncha, the Phthiraptera still fell inside ingroup and were in a sister clade to the Fulgoroidea.

**Figure 5:**
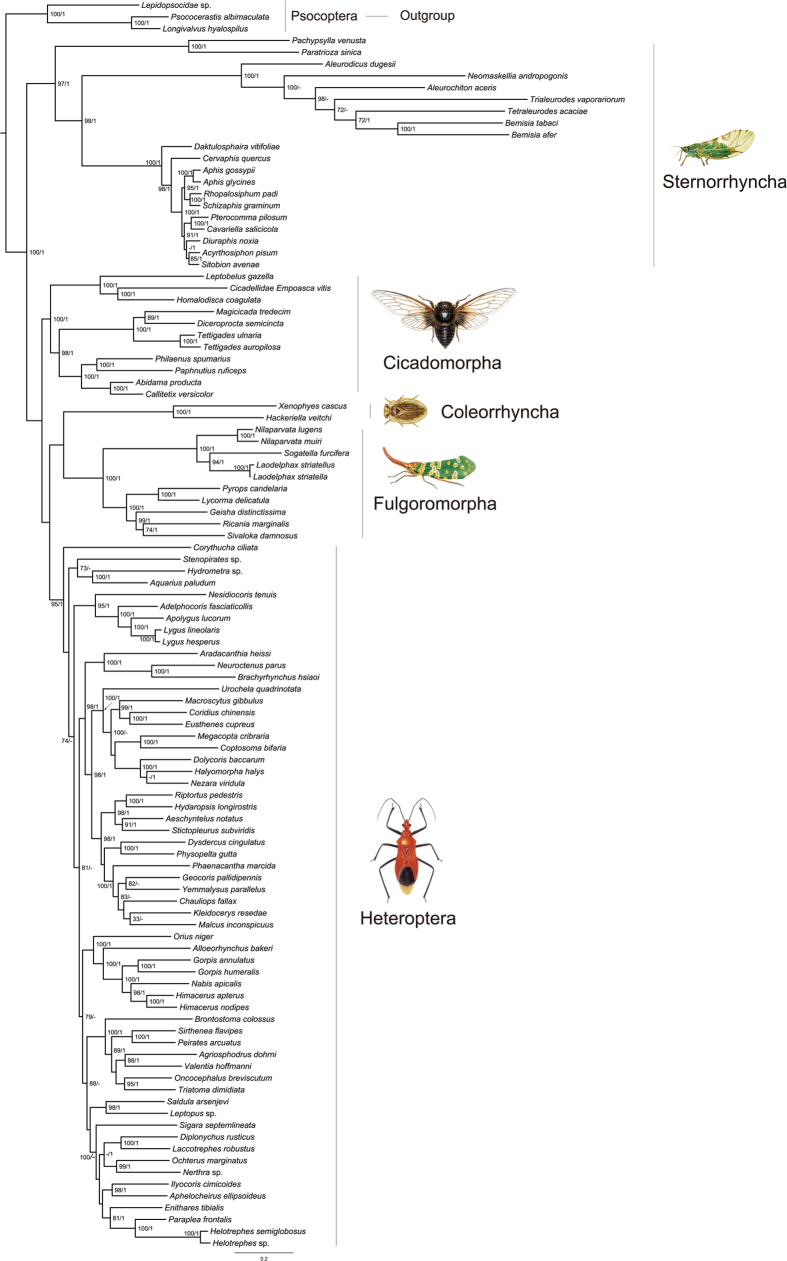
Maximum likelihood tree estimated from the reduced dataset of 106taxa_PCGRNA using data partitions and model selections as in Table S3. MrBayes analysis recovered a similar topology to ML analysis. Node numbers show bootstrap support values (above 70, right) and posterior probabilities (above 0.9, left). The scale bar represents substitutions/site.

The weak support for key nodes and frequent conflicts between different analyses based on the above-mentioned concatenate datasets led us to do further analyses. Long branches occurred in all datasets, in particular with regard to the outgroup clades Thysanoptera, Phthiraptera, Liposcelididae and the ingroup Aleyrodidae (Sternorrhyncha). Thus, in order to reduce the effect of long-branch attraction, we excluded the Thysanoptera, Phthiraptera, and Liposcelididae to compile a reduced dataset of 106taxa_PCGRNA. Based on this dataset, ML and MrBayes analyses recovered a monophyletic Hemiptera, and supported the Sternorrhyncha as a sister group to the remaining Hemiptera (BP = 100, and PP = 1) (Fig. 5). The position of clade (Coleorrhyncha + Fulgoromorpha) changed dramatically with comparison of Fig. 4, and they together grouped with the Heteroptera. However, this relationship received no statistical support. The Phylobayes analysis on the basis of 106taxa_PCGRNA recovered a different relationships within Hemiptera, namely that the cicadas diverged firstly and the clade (Coleorrhyncha + (Fulgoromorpha + Sternorrhyncha)) formed a sister group to Heteroptea. But the latter sister group relationship was weakly supported (PP = 0.7).

The topology test indicated that the ingroup relationships depicted in Fig. 5 represented the most likely Hemiptera phylogeny, and other hypotheses were confidently rejected (Table 4). On the basis of likelihood scores, the ingroup topology of Fig. 5 was most similar to the hypothesis of Fig. 4. For the remaining alternative hypotheses, the Hemiptera phylogenetic relationships inferred by the study of Misof *et al.*^14^ (Fig. 1F) had the highest likelihood scores across all tests. This indicated that our phylogenetic inference of Hemiptera was more similar to that of Misof *et al.*^14^ than others.

## Discussion

### Mitogenome characteristics of *R. padi*

With the rapidly advanced sequencing technology, transcriptomes are now easier to obtain using RNA-seq. Based on the transcriptome data, relatively few primers (13 primers in this study) were used to amplify short gaps mainly in the region of tRNA genes. Whereas, conventional method based on primer walking strategy required 40–50 primers to complete a typical insect mitogenome^55^. This study demonstrated the feasibility of using RNA-seq and gap-filling sequencing for *de novo* assembly of insect mitogenome. The mitogenome of *R. padi* examined in this study has the similar gene content, order, and structure to other published aphid mitogenomes^26^,^27^,^30^,^31^. For the mitochondrial PCGs, the presence of incomplete stop codons is a common phenomenon found in insect mitogenome including the published aphid mitogenomes, for example the *Diuraphis noxia*^30^. A common interpretation for this phenomenon is that the complete termination codon is created by polyadenylation of mRNA^56^. In this study, the poly(A) stretches were found in the 3′ end of *cox1* and *nad4* transcripts, which might be critical to generate the complete termination codon^57^. In the case of *trnS(AGN)* of *R. padi*, the dihydrouridine (DHU) arm cannot form, as in many other insect species^58^. The gene length and base composition of two *R. padi* rRNAs are similar other aphid species^26^,^27^,^30^,^31^.

### Strategies to ameliorate long-branch attraction artifact

Sequences of the mitogenome have been extensively used for inferring phylogenetic relationships at different taxonomic levels^59^. In particular, they have been widely used for deciphering intraordinal relationships within insects^12^,^60^,^61^. Due to mutational saturation, heterogeneity in nucleotide composition, and lineage-specific rate acceleration, the usefulness of mitogenome as a marker for higher level insect systematics remains controversial^62^. Some insects within Paraneoptera have been shown to be having accelerated substitution rates and significant saturation on the third codon positions^13^,^31^,^63^. In the phylogenetic reconstruction, these insects usually display extremely long-branch length, which have the potential to cause the long-branch attraction artifacts (LBA)^13^,^64^. Within Hemitptera, the Aleyrodidae (Sternorrhyncha) showed the longest branches (Table 1, branch lengths of the Sternorrhyncha are represented by the longest whitefly), and consistently clustered together with long-branched outgroups Thysanoptera, Phthiraptera and Lepidopsocidae, particularly in analyses using homogeneous model. This result may be due to their shared nucleotide compositional biases as shown by the high GC-skew values (Fig. 3 and Fig. S1). Here we applied various sequence coding strategies and model settings to reduce the impact of long-branch attraction. From the analysis of 13 different datasets with full taxa (i.e., rRNAs, tRNAs, PCG123, PCG_AA, 7gene_AA, PCG12, PCG3RY, PCG1RY2nd, PCGDegen, PCGRNA, PCG12RNA, PCG1RY2ndRNA, and PCGDegenRNA) under homogeneous model, only the rRNA sequences recovered the Hemiptera as monophyletic (Table 1). A previous study had suggested that different mitochondrial genes have distinct rates of molecular evolution^65^. According to Mueller (2006), two mitochondrial rRNAs (*rrnS* and *rrnL*) have the slowest rates of evolution^65^. Thus, our results indicated that slowly evolving genes were relatively immune to LBA artifacts, and might be preferred loci for resolving deep-level relationships in Hemiptera. In addition, increased taxon sampling is necessary to break up long branches. Previous phylogenetic studies of Hemiptera have shown that the aphid is one of the closet relatives of whiteflies^9^,^10^,^14^,^15^,^18^. Although they are both the stemorrhynchans with similar biological characters, such as faster generation times and more generation, yet the aphids’ mitogenomes exhibit lower sequence evolutionary rate and shorter branch lengths than whiteflies^12^,^15^,^31^. Thus, addition of aphid mitogenome data has the potential to alleviate the long-branch effect caused by whiteflies, and to increase the accuracy of phylogenetic estimation of Hemiptera.

Among various data treatments, Degen-coding strategy may be the most effective method of suppressing long-branch attraction (Fig. S2). Degen-coding was designed to reduce nucleotide compositional heterogeneity and improve resolution of deep-level arthropod relationships^40^,^41^. This approach eliminates all synonymous changes by extending other coding schemes (e.g., RY-coding) to degenerate all codons completely. At the same time, all nonsynonymous changes are retained at the third codon position. This is the advantage of this type of data treatment method compared with other data coding schemes. Because the often used data coding strategy of completely removing sites pays a very high cost of reduction in resolution of phylogenetic relationships^66^. In the trees inferred from Degen-coding datasets (PCGDegen and PCGDegenRNA), the long-branch attraction between ougroup and ingroup was significantly reduced. Moreover, for the hypothesis testing, Degen-coding datasets showed greater likelihood scores (Table 4). This also demonstrated the benefit of Degen-coding strategy to Hemitpera phylogenetic inference. In addition, our analyses indicated that the site-heterogeneous model can mediate long-branch effects and recover a rational Hemitpera phylogeny. The fit of the heterogeneous model to paraneopteran mitogenomic data over the homogeneous model was also suggested in prior study by Li *et al.*^13^. Finally, in order to obtain a well-resolved Hemiptera phylogeny from current mitogenomic data, the long-branch outgroup taxa were excluded to overcome LBA artifacts. This approach had a drastic effect in the tree reconstruction, where Hemiptera were recovered by all optimal criteria. And the Sternorrhyncha was strongly supported as the earliest splitting lineage in Hemiptera (Fig. 5).

### Mitogenome-based phylogeny of Hemiptera

From the point of view of avoiding LBA artifacts, the Hemiptera relationships illustrated in Fig. 5 are preferred as the best estimation. This hypothesis was further corroborated by the topology test (Table 4). On the basis of this tree, the monophyly of Hemiptera was strongly supported. Sternorrhyncha was placed as the sister group to all other Hemiptera, rendering Homptera paraphyletic. This arrangement is in concordance with most recent phylogenetic studies on Hemiptera^7^,^8^,^9^,^10^,^12^,^13^,^14^. Despite these, we should acknowledge the limitations of mitochondrial data in solving the deep-level phylogeny of Hemiptera, in particular some deepest nodes of the tree in this study do not receive good support. Mitogenome is a kind of rapidly evolving gene locus. In particular, contrasting rates of mitogenomes among paraneopteran insects resulted in significantly uneven branch length on phylogenetic trees (Table 1). This confounded the reconstruction of hemipteran relationships. If sequence compositional heterogeneity is not considered, simultaneously including long-branched outgroup and ingroup taxa in a phylogenetic analysis must introduce LBA artifacts. Under this situation, applying appropriate data treatments can improve phylogenetic results of Hemiptera. This study has shown that some recoding strategies can reduce the degree of compositional heterogeneity and substitution rates of mitogenome sequences. When the Degen-coding or RY-coding schemes were used to phylogenetic analyses, better resolved hemipteran trees were inferred from the full taxa datasets under the site-heterogeneous model.

The Cicadomorpha was placed next to Sternorrhyncha in the ML tree from 106taxa_PCGRNA (Fig. 5). For the remaining hemipterans, a sister-group of Coleorrhyncha and Fulgoromorpha formed a clade, which is sister to Heteroptea. This result appears anomalous, but it is in agreement with the fossil evidence that fulgoromorphs arose independently of a polyphyletic Cicadomorpha at the end of the early Permian^67^. The close relationship between Coleorrhyncha and Fulgoromorpha was recovered by all analyses in the present study. This arrangement largely concurred with the results from Cui *et al.*^12^ and Misof *et al.*^14^. However, it rejected the hypothesis of Heteropterodea^7^,^8^,^9^,^10^,^18^,^19^. The paleontological record indicated no evidence of an immediate common ancestor of Coleorhyncha and Heteroptera, and they originated independently from separate lineages of auchenorrhynchan ingruids^68^. The Auchenorrhyncha is another controversial problem of Hemiptera phylogeny. Most molecular studies suggested Fulgoromorpha and Cicadomorpha were likely to be separate lineages occupying independent positions within Hemiptera^9^,^10^,^69^. This conclusion led to a non-monophyletic Auchenorrhyncha. Conversely, in some analyses^18^,^70^, Auchenorrhyncha was supported as a monophyletic group. Although the phylogenetic affiliations inferred by dataset of 106taxa_PCGRNA did not support the Auchenorrhyncha, three other analyses (122taxa_rRNAs RAxML, 122taxa_PCGDegen PhyloBayes and 122taxa_PCGRNA PhyloBayes) recovered a group of Auchenorrhyncha including Coleorhyncha in this study. Thus, the monophyly of Auchenorrhyncha deserved to be further tested by more mitogenome data from homopteran insects.

As a whole, the preferred hemipteran genealogy at suborder and infraorder levels reconstructed on the basis of current mitogenomic data are more similar to Misof *et al.*^14^. Both studies supported the most basal placement of Sternorrhyncha, and a closer relationship of Coleorhyncha to Fulgoromorpha than Heteroptera.

### Sequence and original tree files

Sequence and original tree files constructed in this article are available in the TreeBASE http://purl.org/phylo/treebase/phylows/study/TB2:S18206.

## Additional Information

**How to cite this article**: Song, N. *et al.* Application of RNA-seq for mitogenome reconstruction, and reconsideration of long-branch artifacts in Hemiptera phylogeny. *Sci. Rep.* **6**, 33465; doi: 10.1038/srep33465 (2016).

## Supplementary Material

Supplementary Information

## Figures and Tables

**Table 1:** Nodal supports and branch lengths for major lineages in each tree.

Dataset	Psocoptera excluding *Liposcelis*		Thysanoptera		Phthiraptera including *Liposcelis*		Hemiptera		Homoptera incuding Coleorrhyncha		Sternorrhyncha		Auchenorrhyncha including Coleorrhyncha		Coleorrhyncha		Heteroptera		Aphidoidea	
	NS	BL	NS	BL	NS	BL	NS	BL	NS	BL	NS	BL	NS	BL	NS	BL	NS	BL	NS	BL
RAxML analyses																				
122taxa_rRNAs	94	0.53	100	1.11	67	1.27	98	1.18	—	—	<50	1.18	56	0.77	68	0.65	100	0.71	100	0.49
122taxa_tRNAs	89	0.52	100	1.61	81	2.40	—	—	—	—	—	—	—	—	95	0.88	100	1.03	100	0.59
122taxa_PCG123	100	2.16	100	5.16	100	9.16	—	—	—	—	56	7.64	—	—	100	3.28	100	4.06	100	3.95
122taxa_PCG_AA	100	0.81	100	2.13	100	3.47	—	—	—	—	<50	3.31	—	—	100	1.41	100	1.76	100	1.81
122taxa_7gene_AA	100	0.59	100	1.92	100	2.84	—	—	—	—	<50	2.63	—	—	99	0.98	100	1.38	100	1.54
122taxa_PCG12	100	0.31	100	0.72	100	1.40	—	—	—	—	<50	1.17	—	—	100	0.52	100	0.57	100	0.57
122taxa_PCG3RY	100	0.93	100	2.45	100	4.37	—	—	—	—	—	—	—	—	100	1.46	100	1.76	100	1.87
122taxa_PCG1RY2nd	100	0.35	100	1.00	100	1.74	—	—	—	—	<50	1.53	—	—	100	0.57	100	0.73	100	0.80
122taxa_PCGDegen	100	0.50	100	1.47	100	2.49	—	—	—	—	53	2.02	—	—	100	0.89	100	1.00	100	1.12
122taxa_PCGRNA	100	0.51	100	1.14	100	1.77	—	—	—	—	97	1.74	—	—	99	0.68	100	0.83	100	0.75
122taxa_PCG12RNA	100	0.35	100	0.86	100	1.42	—	—	—	—	<50	1.14	—	—	<50	0.52	100	0.60	100	0.53
122taxa_PCG1RY2ndRNA	100	0.43	100	1.19	100	1.69	—	—	—	—	—	—	—	—	—	—	100	0.71	100	0.93
122taxa_PCGDegenRNA	100	0.36	100	0.94	100	1.53	—	—	—	—	77	1.25	—	—	79	0.55	100	0.63	100	0.60
106taxa_PCGRNA	100	0.49	—	—	—	—	100	1.79	—	—	97	1.79	—	—	95	0.78	100	0.92	100	0.69
MrBayes analyses																				
122taxa_rRNAs	1.00	0.10	1.00	0.19	1.00	0.19	1.00	0.24	1.00	0.24	1.00	0.24	—	—	1.00	0.10	1.00	0.08	1.00	0.12
122taxa_tRNAs	1.00	0.34	1.00	1.17	1.00	1.54	—	—	—	—	—	—	—	—	1.00	0.69	1.00	0.44	1.00	0.28
122taxa_PCG123	1.00	1.69	1.00	3.04	1.00	7.18	—	—	—	—	1.00	6.38	—	—	1.00	2.82	2.09		1.00	3.40
122taxa_PCG_AA	1.00	0.40	1.00	1.00	1.00	1.45	—	—	—	—	1.00	1.43	—	—	1.00	0.73	1.00	0.72	1.00	0.84
122taxa_7gene_AA	1.00	0.28	1.00	0.75	1.00	0.97	—	—	—	—	1.00	1.11	—	—	1.00	0.58	1.00	0.45	1.00	0.67
122taxa_PCG12	1.00	0.33	1.00	0.91	1.00	1.53	—	—	—	—	—	—	—	—	1.00	0.66	1.00	0.51	1.00	0.75
122taxa_PCG3RY	1.00	0.72	1.00	1.71	1.00	2.98	—	—	—	—	0.97	2.83	—	—	1.00	1.27	1.00	0.94	1.00	1.48
122taxa_PCG1RY2nd	1.00	0.53	1.00	1.46	1.00	2.31	—	—	—	—	—	—	—	—	1.00	0.93	1.00	0.82	1.00	1.19
122taxa_PCGDegen	1.00	0.46	1.00	1.37	1.00	2.25	—	—	—	—	<0.90	2.22	—	—	1.00	0.89	1.00	0.80	1.00	1.14
122taxa_PCGRNA	1.00	1.41	1.00	3.37	1.00	5.09	—	—	—	—	1.00	4.74	—	—	1.00	2.09	1.00	1.80	1.00	2.45
122taxa_PCG12RNA	1.00	0.55	1.00	1.41	1.00	2.02	—	—	—	—	—	—	—	—	1.00	0.94	—	—	1.00	1.25
122taxa_PCG1RY2ndRNA	1.00	0.03	1.00	0.09	1.00	0.14	—	—	—	—	—	—	—	—	1.00	0.06	—	—	1.00	0.08
122taxa_PCGDegenRNA	1.00	0.24	1.00	0.69	1.00	0.95	—	—	—	—	—	—	—	—	1.00	0.43	<0.90	0.44	1.00	0.46
106taxa_PCGRNA	1.00	0.50	—	—	—	—	1.00	2.00	—	—	1.00	2.00	—	—	1.00	0.79	1.00	0.83	1.00	0.93
PhyloBayes analyses																				
122taxa_rRNAs	0.98	1.23	1.00	3.19	<0.90	3.63	<0.90	3.42	<0.90	3.42	<0.90	3.42	—	—	1.00	1.19	1.00	1.37	<0.90	1.48
122taxa_tRNAs	0.99	0.72	1.00	3.24	<0.90	6.33	—	—	—	—	—	—	—	—	1.00	1.50	1.00	1.21	<0.90	0.91
122taxa_PCG123	1.00	1.04	1.00	2.83	<0.90	6.38	—	—	—	—	<0.90	5.70	—	—	1.00	1.76	<0.90	1.19	<0.90	2.42
122taxa_PCG_AA	1.00	0.67	1.00	1.26	1.00	5.28	—	—	—	—	<0.90	4.42	—	—	1.00	1.56	1.00	1.28	1.00	2.22
122taxa_7gene_AA	1.00	0.49	1.00	1.68	1.00	3.44	—	—	—	—	0.93	2.21	—	—	1.00	1.35	1.00	1.15	1.00	1.78
122taxa_PCG12	<0.90	0.54	1.00	2.11	<0.90	4.96	—	—	—	—	<0.90	3.52	—	—	1.00	1.12	0.90	0.93	1.00	1.73
122taxa_PCG3RY	0.92	0.52	1.00	2.24	0.95	5.05	—	—	—	—	<0.90	3.65	—	—	1.00	1.21	1.00	0.93	1.00	1.83
122taxa_PCG1RY2nd	1.00	0.50	1.00	1.93	1.00	4.24	—	—	—	—	<0.90	2.53	—	—	1.00	0.95	1.00	0.86	1.00	1.46
122taxa_PCGDegen	—	—	1.00	1.78	<0.90	5.36	—	—	—	—	0.91	3.23	<0.90	1.38	1.00	0.96	0.96	0.80	1.00	1.37
122taxa_PCGRNA	1.00	0.94	1.00	2.88	<0.90	7.25	—	—	—	—	<0.90	5.02	<0.90	1.92	1.00	1.59	<0.90	1.13	1.00	1.93
122taxa_PCG12RNA	1.00	0.71	1.00	2.50	1.00	5.47	—	—	—	—	1.00	3.94	—	—	1.00	1.26	1.00	1.06	1.00	1.65
122taxa_PCG1RY2ndRNA	0.99	0.63	1.00	2.64	1.00	4.88	<0.90	2.12	—	—	—	—	—	—	1.00	1.17	—	—	1.00	1.39
122taxa_PCGDegenRNA	1.00	0.64	1.00	2.40	1.00	5.57	—	—	—	—	1.00	3.64	—	—	1.00	1.14	1.00	0.96	1.00	1.54
106taxa_PCGRNA	1.00	0.97	—	—	—	—	1.00	5.80	—	—	1.00	5.80	—	—	1.00	1.79	1.00	1.43	1.00	2.29

The branch lengths were calculated from the longest terminal taxon of each lineage to the common ancestor to the Paraneoptera.

“—” denote the monophyletic lineage not to be recovered by the dataset.

NS: nodal support; BL: branch length.

**Table 2:** Annotation of *Rhopalosiphum padi* mitogenome.

Locus Name	Start	Stop	Strand	Length	Start codon	Stop codon	Anticodon	Intergenic nucleotide
*trnI*	1	64	+	64			GAT	−
*trnQ*	62	127	−	66			TTG	−3
*trnM*	136	201	+	66			CAT	8
*nad2*	202	1179	+	975	ATA	TAA		0
*trnW*	1178	1240	+	63			TCA	−2
*trnC*	1233	1301	−	69			GCA	−8
*trnY*	1304	1370	−	67			GTA	2
*cox1*	1372	2901	+	1530	ATA	T−		1
*trnL(UUR)*	2903	2970	+	68			TAA	1
*cox2*	2974	3645	+	672	ATA	TAA		3
*trnK*	3648	3720	+	73			CTT	2
*trnD*	3721	3782	+	62			GTC	0
*atp8*	3783	3941	+	159	ATT	TAA		0
*atp6*	3922	4575	+	654	ATT	TAA		−20
*cox3*	4575	5360	+	783	ATG	TAA		−1
*trnG*	5360	5423	+	64			TCC	−1
*nad3*	5424	5777	+	357	ATT	TAA		0
*trnA*	5778	5841	+	64			TGC	0
*trnR*	5841	5905	+	65			TCG	−1
*trnN*	5905	5971	+	67			GTT	−1
*trnS(AGN)*	5971	6032	+	62			GCT	−1
*trnE*	6035	6099	+	65			TTC	2
misc_feature	6100	6181		82				0
misc_feature	6182	6248		67				0
*nad5*	6249	7913	−	1665	ATA	T−		0
*trnH*	7914	7977	−	64			GTG	0
*nad4*	7975	9283	−	1308	ATA	T−		0
*nad4l*	9283	9573	−	288	ATA	TAA		−1
*trnT*	9575	9636	+	62			TGT	1
*trnP*	9639	9704	−	66			TGG	2
*nad6*	9706	10200	+	486	ATT	TAA		1
*cob*	10200	11315	+	1116	ATG	TAG		−1
*trnS(UCN)*	11314	11378	+	65			TGA	−2
*nad1*	11389	12279	−	888	ATA	TAA		10
*trnL(CUN)*	12286	12350	−	65			TAG	6
*rrnL*	12351	13609	−	1259				0
*trnV*	13610	13671	−	62			TAC	0
*rrnS*	13674	14440	−	767				2
CR region	14441	15205		764				0

Note: “+” indicates major strand, “−” indicates minor strand.

**Table 3:** Substitution saturation tests.

Data partition	NumOTU	*Iss*	*Iss.cSym*^a^	*Psym*^b^	*Iss.cAsym*^c^	*Pasym*^d^
PCG123	16	0.593	0.850	0.000	0.676	0.000
	32	0.603	0.818	0.000	0.572	0.000
PCG12	16	0.485	0.841	0.000	0.681	0.000
	32	0.496	0.814	0.000	0.570	0.000
PCG1	16	0.566	0.827	0.000	0.668	0.000
	32	0.576	0.808	0.000	0.553	0.000
PCG2	16	0.419	0.827	0.000	0.668	0.000
	32	0.430	0.808	0.000	0.553	0.000
PCG3	16	0.880	0.827	0.000	0.668	0.000
	32	0.884	0.808	0.000	0.553	0.000
tRNAs	16	0.594	0.790	0.000	0.605	0.000
	32	0.615	0.772	0.000	0.486	0.000
rRNAs	16	0.614	0.806	0.000	0.633	0.000
	32	0.639	0.79	0.000	0.520	0.000

^a^Index of substitution saturation assuming a symmetrical true tree.

^b^Probability of significant difference between Iss and Iss.cSym (two-tailed test).

^c^Index of substitution saturation assuming an asymmetrical true tree.

^d^Probability of significant difference between Iss and Iss.cAsym (two-tailed test).

**Table 4:**
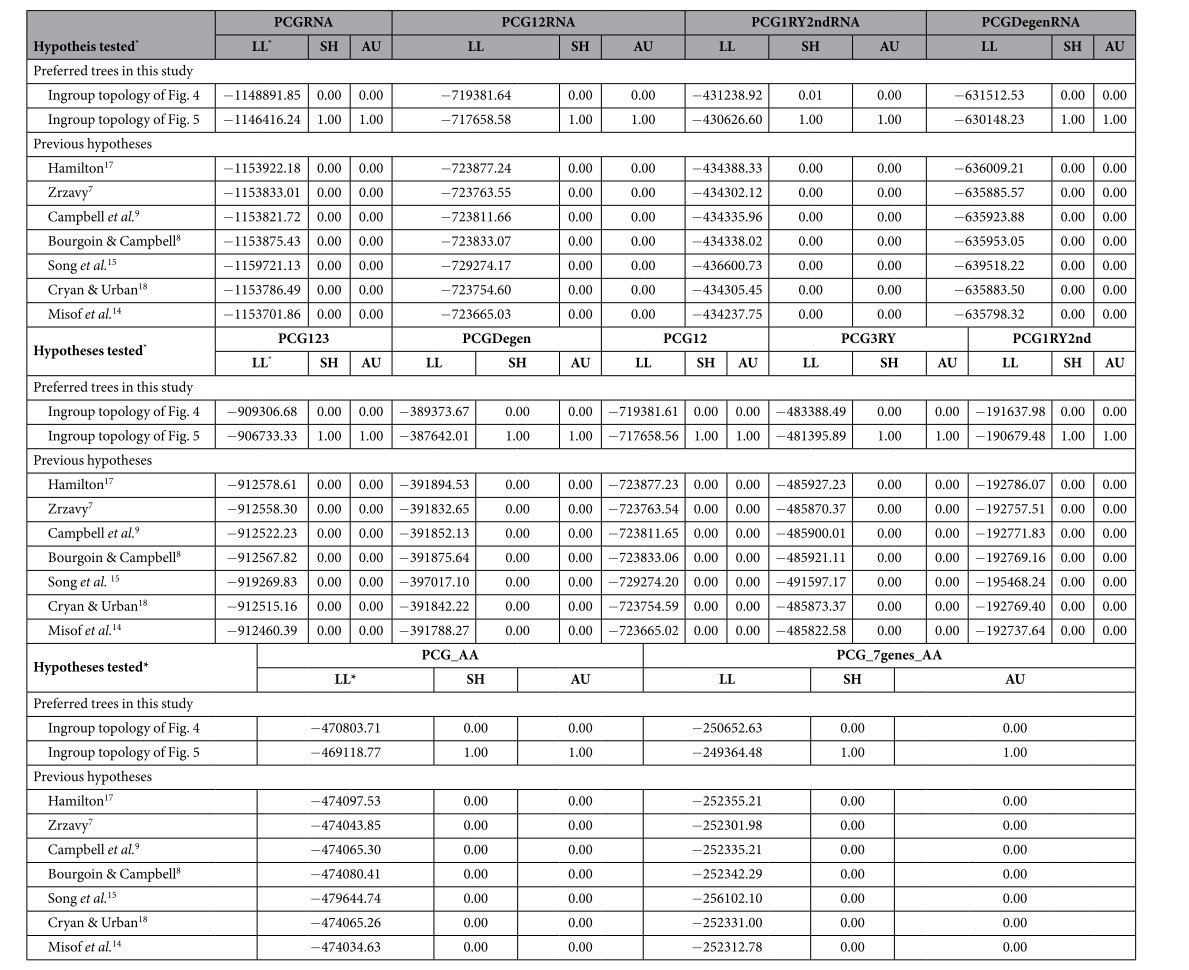
Hypothesis testing.

*LL: Log-likelihood.

*Hypothesis tested are the following.

Hamilton^17^: ((Heteroptera, Coleorhyncha), (Fulgoromorpha, (Sternorrhyncha, Cicadomorpha))).

Song *et al.*^15^: ((Cicadomorpha, (Fulgoromorpha, Sternorrhyncha)), Heteroptera).

Zrzavy^7^: (Sternorrhyncha, ((Cicadomorpha, Fulgoromorpha), (Heteropteara, Coleorrhyncha))).

Campbell *et al.*^9^: (Sternorrhyncha, (Cicadomorpha, (Fulgoromorpha, (Heteropteara, Coleorrhyncha)))).

Bourgoin & Campbell^8^: (Sternorrhyncha, (Fulgoromorpha, (Cicadomorpha, (Heteropteara, Coleorrhyncha)))).

Cryan & Urban^18^: (Sternorrhyncha, ((Heteropteara, Coleorrhyncha), (Fulgoromorpha, Cicadomorpha))).

Misof *et al.*^14^: (Sternorrhyncha, (Heteroptera, (Coleorrhyncha, (Fulgoromorpha, Cicadomorpha))))).
