# Supplementary material for: Application of RNA-seq for mitogenome reconstruction, and reconsideration of long-branch artifacts in Hemiptera phylogeny
Source: Sci Rep. 2016 Sep 16;6:33465. doi: 10.1038/srep33465 (PMC5025853; doi:10.1038/srep33465)
Supplement: Supplementary Information [file srep33465-s1.pdf]

Application of RNA-seq for mitogenome reconstruction, and reconsideration of long-branch artifacts in Hemiptera phylogeny

Nan Song[1, *], Shiheng An[1], Xinming Yin[1], Wanzhi Cai[2], Hu Li[2]

1 College of Plant Protection, Henan Agricultural University, Zhengzhou, China,

2 Department of Entomology, China Agricultural University, Beijing, China

* E-mail: songnan@henau.edu.cn

Table S1 Species-specific primers designed for amplification of remaining mitochondrial gene from *Rhopalosiphum padi* DNA.

N2-J586	CCTTTTCACTTATGATTACC	C1-N2353	GCTCGTGTATCAACGTCTATTCC
C1-J2195	TGATTTTTTGGACATCCAGAAGT	C2-N3389	TATTCATATGATCAGAATCATTG
C2-J3624	ATCCCAGGACGAATAAATCA	A6-N4552	ATGGCCTGCAATTAAATTAGC
A6-J4463	ATTGCACACTTGATTCCTTTAAA	C3-N4908	CGAATTACATCTCGTCATCATAA
C3-J5470	TTAGGAGCTTGATATTGACA	N3-N5731	AATGGGTCAAAACCACATTC
N3-J5747	CCCTTTGAATGTGGTTTTGACCC	N5-N7211	TTAAATCTATAATATTTTTAAGAGC
TN_J6172	AGAGGTTTATTACTGTTAATAA	N5-N7211	identical to above
N5-J7572	TGTTTGACAATATTAATAGCTGGT	N4-N8487	TCCCAGCTAAAATTATTGAACC
N4-J9172	GGCTCATGTTGAGGCTCCTA	CB-N11010	TATTAATTGAAAATCCTCCTCA
CB-J11335	CACATTCAACCAGAATGATA	LR-N13000	TTACTTTAGGGATAACAGCGTAA
LR-J12888	CCGGTTTGAACTCAAATCATGTA	SR-N14220	ATATGTACATATTGCCCGTC
SR-J14197	GTACATTTACTTTGTTACGACTT	SR-N14745	GTGCCAGCAGTTGCGGTTAAAC
SR-J14610	ATAATAGGGTATCTAATCCTRGT	TM-N200	ACCTTTATAARTGGGGTATGARCC

Table S2. Taxonomic information and GenBank accession numbers for the taxa included in this study.

Higher Taxon	Superfamily	Family	Species	Accession number
Cimicomorpha	Membracoidea	Cicadellidae	*Homalodisca coagulata*	AY875213
			Leptobelus gazella	NC_023219
			Empoasca vitis	NC_024838
	Cercopoidea	Aphrophoridae	*Philaenus spumarius*	AY630340
		Cercopidae	*Callitetix versicolor*	EU725832
			Abidama producta	NC_015799
			Paphnutius ruficeps	NC_021100
	Cicadoidea	Cicadidae	*Magicicada tredecim*	KM000130
			Tettigades auropilosa	KM000129
			Tettigades ulnaria	KM000128
			Diceroprocta semicincta	KM000131
Fulgoromorpha	Fulgoroidea	Delphacidae	*Laodelphax striatellus*	FJ360695
			Laodelphax striatella	JX880068
			Nilaparvata lugens	JN563995
			Sogatella furcifera	NC_021417
			Nilaparvata muiri	NC_024627
		Fulgoridae	*Lycorma delicatula*	EU909203
			Pyrops candelaria	FJ006724
		Ricaniidae	*Ricania marginalis*	JN242415
		Flatidae	*Geisha distinctissima*	FJ230961
		Issidae	*Sivaloka damnosus*	FJ360694
Coleorrhyncha	Peloridioidea	Peloridiidae	*Xenophyes cascus*	JF323862
			Hackeriella veitchi	NC_020309
Sternorrhyncha	Psylloidea	Psyllidae	*Pachypsylla venusta*	AY263317
		Triozidae	*Paratrioza sinica*	KJ650081
	Aphidoidea	Aphididae	*Schizaphis graminum*	AY531391
			Acyrthosiphon pisum	FJ411411
			Aphis glycines	KC840675
			Aphis gossypii	NC_024581
			Diuraphis noxia	NC_022727
			Pterocomma pilosum	KC840676
			Rhopalosiphum padi	**KT447631**
			Sitobion avenae	NC_024683
			Cavariella salicicola	NC_022682
		Greenideidae	*Cervaphis quercus*	NC_024926
		Phylloxeridae	*Daktulosphaira vitifoliae*	DQ021446
	Aleyrodoidea	Aleyrodidae	*Aleurodicus dugesii*	AY521251
			Neomaskellia andropogonis	AY572539
			Trialeurodes vaporariorum	AY521265
			Tetraleurodes acaciae	AY521262
			Aleurochiton aceris	AY572538
			Bemisia afer	KF734668
			Bemisia tabaci	AY521259
Heteroptera	Cimicoidea	Alydidae	*Riptortus pedestris*	EU427344
		Anthocoridae	*Orius niger*	EU427341

Aphelocheiroide	Aphelocheiridae	*Aphelocheirus ellipsoideus*	FJ456939
Aradoidea	Aradidae	*Brachyrhynchus hsiaoi*	NC_022670
		Aradacanthia heissi	HQ441233
		Neuroctenus parus	EU427340
Nepoidea	Belostomatidae	*Diplonychus rusticus*	FJ456940
Lygaeoidea	Berytidae	*Yemmalysus parallelus*	EU427346
	Colobathristidae	*Phaenacantha marcida*	EU427342
Cimicoidea	Coreidae	*Hydaropsis longirostris*	EU427337
		Sigara septemlineata	FJ456941
Pentatomoidea	Cydnidae	*Macroscytus subaeneus*	EU427338
	Dinidoridae	*Coridius chinensis*	JQ739179
Enicocephaloide	Enicocephalidae	*Stenopirates* sp.	NC_016017
Ochteroidea	Gelastocoridae	*Nerthra* sp.	FJ456943
Lygaeoidea	Geocoridae	*Geocoris pallidipennis*	EU427336
	Helotrephidae	*Helotrephes semiglobosus semiglobosus*	KJ027513
		Helotrephes sp. NKMT027	FJ456951
Hydrometroidea	Hydrometridae	*Hydrometra* sp.	FJ456945
Pyrrhocoroidea	Largidae	*Physopelta gutta*	EU427343
Leptopodoidea	Leptopodidae	*Leptopus* sp.	FJ456946
Lygaeoidea	Lygaeidae	*Kleidocerys resedae resedae*	KJ584365
	Malcidae	*Chauliops fallax*	NC_020772
		Malcus inconspicuus	EU427339
Miroidea	Miridae	*Lygus hesperus*	NC_024641
		Adelphocoris fasciaticollis	NC_023796
		Apolygus lucorum	NC_023083
		Nesidiocoris tenuis	NC_022677
		Lygus lineolaris	NC_021975
Cimicoidea	Nabidae	*Alloeorhynchus bakeri*	NC_016432
		Gorpis annulatus	NC_019595
		Gorpis humeralis	NC_019593
		Himacerus apterus	JF927831
		Himacerus nodipes	JF927832
		Nabis apicalis	NC_019594
Naucoroidea	Naucoridae	*Ilyocoris cimicoides*	FJ456964
Nepoidea	Nepidae	*Laccotrephes robustus*	FJ456948
Notonectoidea	Notonectidae	*Enithares tibialis*	FJ456949
Ochteroidea	Ochteridae	*Ochterus marginatus*	FJ456950
Pentatomoidea	Pentatomidae	*Dolycoris baccarum*	NC_020373
		Halyomorpha halys	FJ685650
		Nezara viridula	EF208087
	Plataspidae	*Megacopta cribraria*	NC_015342
		Coptosoma bifaria	EU427334
Pleoidea	Pleidae	*Paraplea frontalis*	KJ027516
Pyrrhocoroidea	Pyrrhocoridae	*Dysdercus cingulatus*	EU427335
Reduvioidea	Reduviidae	*Peirates arcuatus*	NC_024264
		Oncocephalus breviscutum	NC_022816
		Triatoma dimidiata	AF301594
		Brontostoma colossus	NC_024745

			Valentia hoffmanni	FJ456952
			Agriosphodrus dohrni	NC_015842
			Sirthenea flavipes	NC_020143
	Cimicoidea	Rhopalidae	*Aeschyntelus notatus*	EU427333
			Stictopleurus subviridis	EU826088
	Saldoidea	Saldidae	*Saldula arsenjevi*	EU427345
	Pentatomoidea	Tessaratomidae	*Eusthenes cupreus*	NC_022449
		Tingidae	*Corythucha ciliata*	NC_022922
	Gerroidea	Gerridae	*Aquarius paludum*	NC_012841
	Pentatomoidea	Urostylididae	*Urochela quadrinotata*	NC_020144
Orthoptera	Pyrgomorphoide	Pyrgomorphidae	*Atractomorpha sinensis*	NC_011824
	Acridoidea	Acrididae	*Locusta migratoria*	NC_001712
			Oxya chinensis	NC_010219
	Tettigonioidea	Tettigoniidae	*Deracantha onos*	NC_011813
			Ruspolia dubia	NC_009876
	Grylloidea	Gryllotalpidae	*Gryllotalpa orientalis*	NC_006678
Psocoptera	Atropetae	Lepidopsocidae	Lepidopsocidae sp.	AF335994
	Nanopsocetae	Liposcelidae	*Liposcelis decolor*	NC_023839
	Psocetae	Psocidae	*Longivalvus hyalospilus*	JQ910986
			Psococerastis albimaculata	NC_021400
Phthiraptera	Philopteroidea	Philopteridae	*Bothriometopus macrocnemis*	NC_009983
			Campanulotes bidentatus compar	NC_007884
			Coloceras sp.	JN122000
			Ibidoecus bisignatus	JN122005
	Boopoidea	Boopidae	*Heterodoxus macropus*	NC_002651
Thysanoptera	Thripoidea	Thripidae	*Frankliniella intonsa*	JQ917403
			Scirtothrips dorsalis	NC_025241
			Frankliniella occidentalis	NC_018370
			Thrips imaginis	AF335993

Note: The mitogenome sequenced in this study is indicated in bold.

Table S3. (A) The partition sechemes and best-fitting models selected by PartitionFinder for dataset of 122-taxa-PCGRNA.

122-taxa-PCGRNA		
**Subset**	**Subset Partitions**	**Best Model**
Partition 1	[trnQ, trnH, trnL(UUR), trnF, trnP]	GTR+G
Partition 2	[trnA, trnI, trnS(UCN), trnY]	GTR+I+G
Partition 3	(trnN, trnD, trnE, trnG, trnT)	GTR+I+G
Partition 4	(cox2_cp1, cox3_cp1, trnK, trnM, trnW)	GTR+I+G
Partition 5	[trnR, atp6_cp1, trnL(CUN), nad3_cp1,trnS(AGN)]	GTR+I+G
Partition 6	(trnC, nad4l_cp1, trnV)	GTR+I+G
Partition 7	(rrnS)	GTR+I+G
Partition 8	(rrnL)	GTR+I+G
Partition 9	(atp6_cp2, cox2_cp2, cox3_cp2, cytb_cp2)	GTR+I+G
Partition 10	(atp6_cp3, cox2_cp3, cox3_cp3, cytb_cp3)	GTR+I+G
Partition 11	(atp8_cp1)	TrN+I+G
Partition 12	(atp8_cp2, nad6_cp2)	GTR+I+G
Partition 13	(atp8_cp3, nad6_cp3)	HKY+I+G
Partition 14	(cox1_cp1)	GTR+I+G
Partition 15	(cox1_cp2)	GTR+I+G
Partition 16	(cox1_cp3)	GTR+I+G
Partition 17	(cytb_cp1)	GTR+I+G
Partition 18	(nad1_cp1, nad4_cp1, nad5_cp1)	GTR+I+G
Partition 19	(nad1_cp2, nad4l_cp2)	GTR+I+G
Partition 20	(nad1_cp3)	GTR+I+G
Partition 21	(nad2_cp1, nad6_cp1)	K81uf+G
Partition 22	(nad2_cp2, nad3_cp2)	TVM+G
Partition 23	(nad2_cp3)	TIM+G
Partition 24	(nad3_cp3)	TrN+I+G
Partition 25	(nad4_cp2, nad5_cp2)	GTR+I+G
Partition 26	(nad4_cp3, nad4l_cp3, nad5_cp3)	TIM+G

Note: "cp_1" representing the first codon position, "cp_2" representing the second codon position, and "cp_3" representing the third codon position.

Abbreviations used in the Best Model: GTR: General-Time-Reversible model; TrN: Tamura-Nei model; HKY: Hasegawa–Kishino–Yano model; K81uf: Kimura three-parameters and unequal base frequencies model; TVM: Tamura–Nei and the transversion model; TIM: transitional model with rAC = rGT ≠ rAT = rCG ≠ rAG ≠ rGT; I: invariant sites; G: discrete Gamma distribution.

Table S3. (B) The partition sechemes and best-fitting models selected by PartitionFinder for dataset of 122-taxa-Amino acids.

122-taxa-Amino acids		
**Subset**	**Subset Partitions**	**Best Model**
Partition 1	(atp6)	MtArt+G+F
Partition 2	(atp8, nad6)	MtREV+I+G+F
Partition 3	(cox1)	MtArt+I+G
Partition 4	(cox2)	MtArt+I+G+F
Partition 5	(cox3)	MtArt+I+G
Partition 6	(cytb)	MtArt+I+G
Partition 7	(nad1)	MtArt+I+G+F
Partition 8	(nad2)	MtREV+G+F
Partition 9	(nad3)	MtREV+G+F
Partition 10	(nad4, nad5)	MtArt+I+G+F
Partition 11	(nad4l)	VT+G+F

Note: "cp_1" representing the first codon position, "cp_2" representing the second codon position, and "cp_3" representing the third codon position.

Abbreviations used in the Best Model: MtArt: the amino acid substitution model with a replacement matrix constructed based on arthropod mt-proteomes; mtREV: Mitochondrial general reversible Markov model; VT: an amino acid substitution model based on the resolvent method, which allows for estimating amino acid substitution from alignments of varying degree of divergence; I: invariant sites; G: discrete Gamma distribution; F: empirical frequencies.

Table S3. (C) The partition sechemes and best-fitting models selected by PartitionFinder for dataset of 106-taxa-PCGRNA.

	**106-taxa-PCGRNA**	
**Subset**	**Subset Partitions**	**Best Model**
Partition 1	[trnR, atp6_cp1,trnL(CUN), nd3_cp1, trnT]	GTR+I+G
Partition 2	(atp6_cp2, cox2_cp2, cox3_cp2, cytb_cp2)	TVM+I+G
Partition 3	(atp6_cp3, atp8_cp3, nad3_cp3)	TrN+G
Partition 4	(atp8_cp1, nad2_cp1, nad6_cp1)	GTR+I+G
Partition 5	(atp8_cp2, nad6_cp2)	GTR+I+G
Partition 6	(cox1_cp1)	GTR+I+G
Partition 7	(cox1_cp2)	TVM+I+G
Partition 8	(cox1_cp3, cox2_cp3, cox3_cp3, cytb_cp3)	GTR+I+G
Partition 9	(cox2_cp1, cox3_cp1, cytb_cp1, trnM)	GTR+I+G
Partition 10	[trnC, trnQ, trnL(UUR), nad1_cp1, trnS(UCN), trnV]	TVM+I+G
Partition 11	(nad1_cp2, nad4_cp2, nad4l_cp2, nad5_cp2)	GTR+I+G
Partition 12	(nad1_cp3, nad5_cp3)	GTR+G
Partition 13	(nad2_cp2, nad3_cp2)	TVM+I+G
Partition 14	(nad2_cp3, nad6_cp3)	TrN+G
Partition 15	(nad4_cp1, nad4l_cp1, nad5_cp1)	GTR+I+G
Partition 16	(nad4_cp3, nad4l_cp3)	TrN+G
Partition 17	(rrnL, rrnS)	GTR+I+G
Partition 18	[trnA, trnK, trnS(AGN)]	TVM+G
Partition 19	(trnN, trnD, trnE,trnI, trnW)	TVM+I+G
Partition 20	(trnG, trnH, trnF, trnP, trnY)	GTR+I+G

Note: "cp_1" representing the first codon position, "cp_2" representing the second codon position, and cp_3 representing the third codon position.

Abbreviations used in the Best Model: GTR: General-Time-Reversible model; TVM: Tamura–Nei and the transversion model; TrN: Tamura-Nei model; I: invariant sites; G: discrete Gamma distribution.

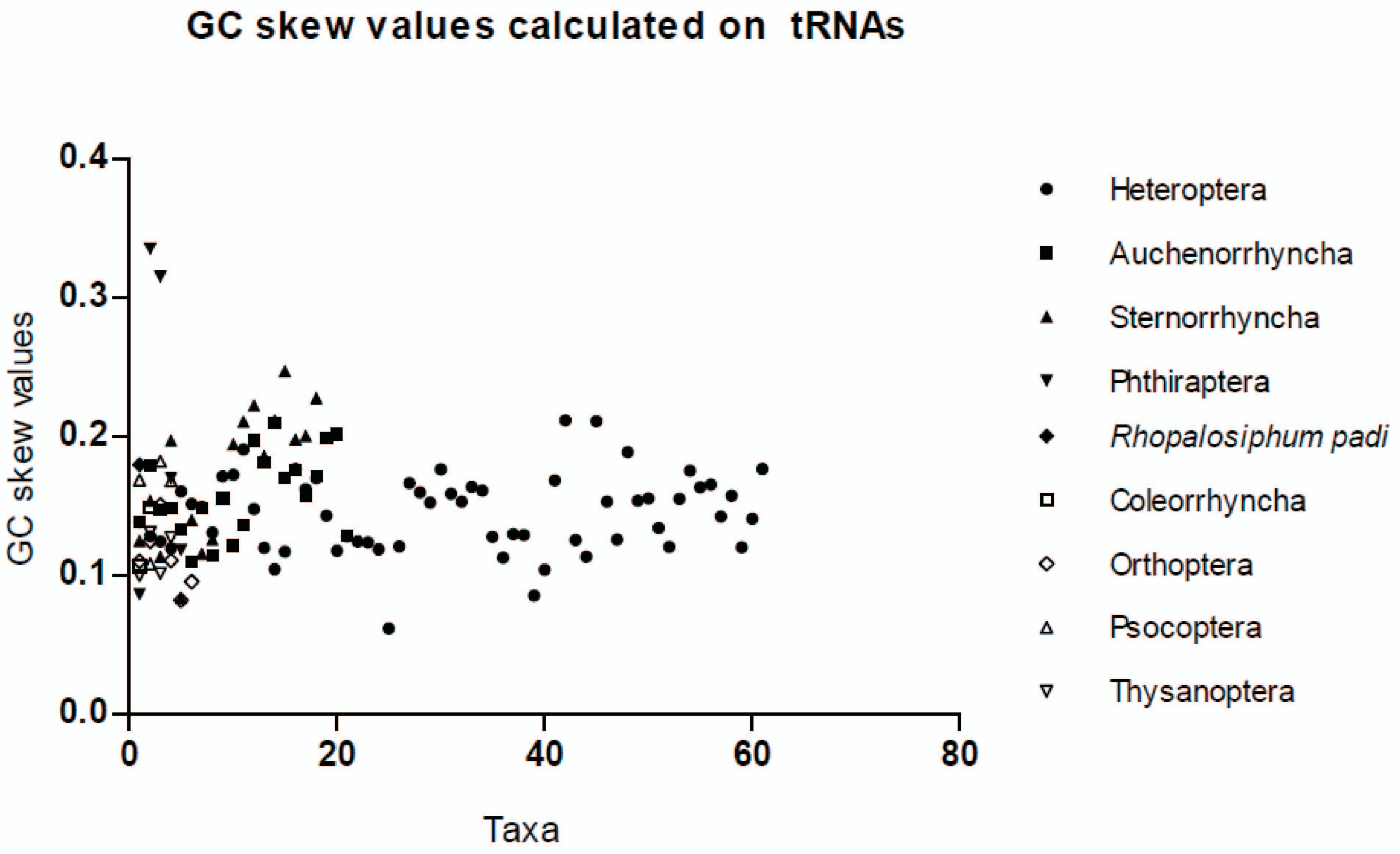

Fig. S1 (A). GC-skew values were calculated on dataset of tRNAs.

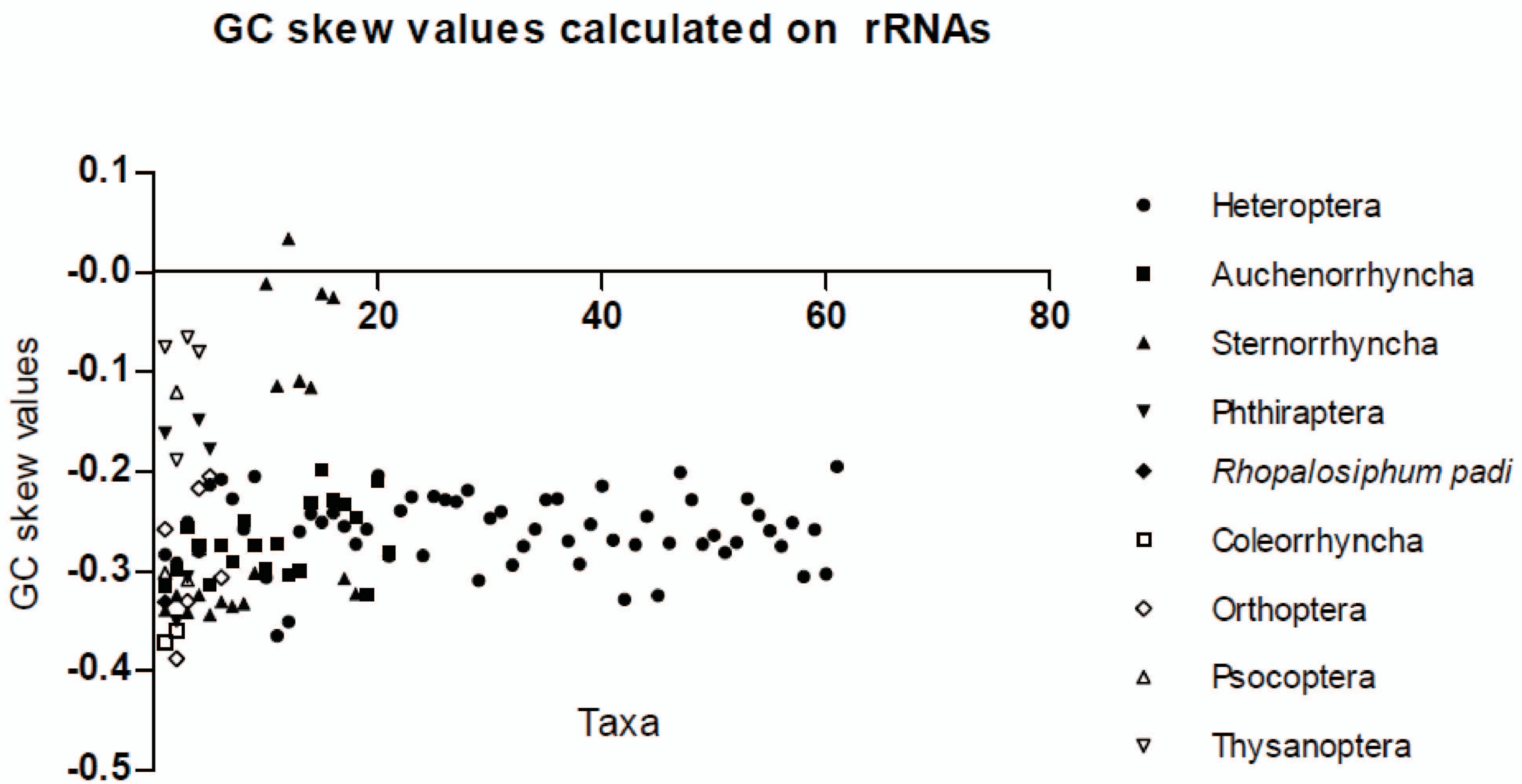

Fig. S1 (B). GC-skew values were calculated on dataset of rRNAs.

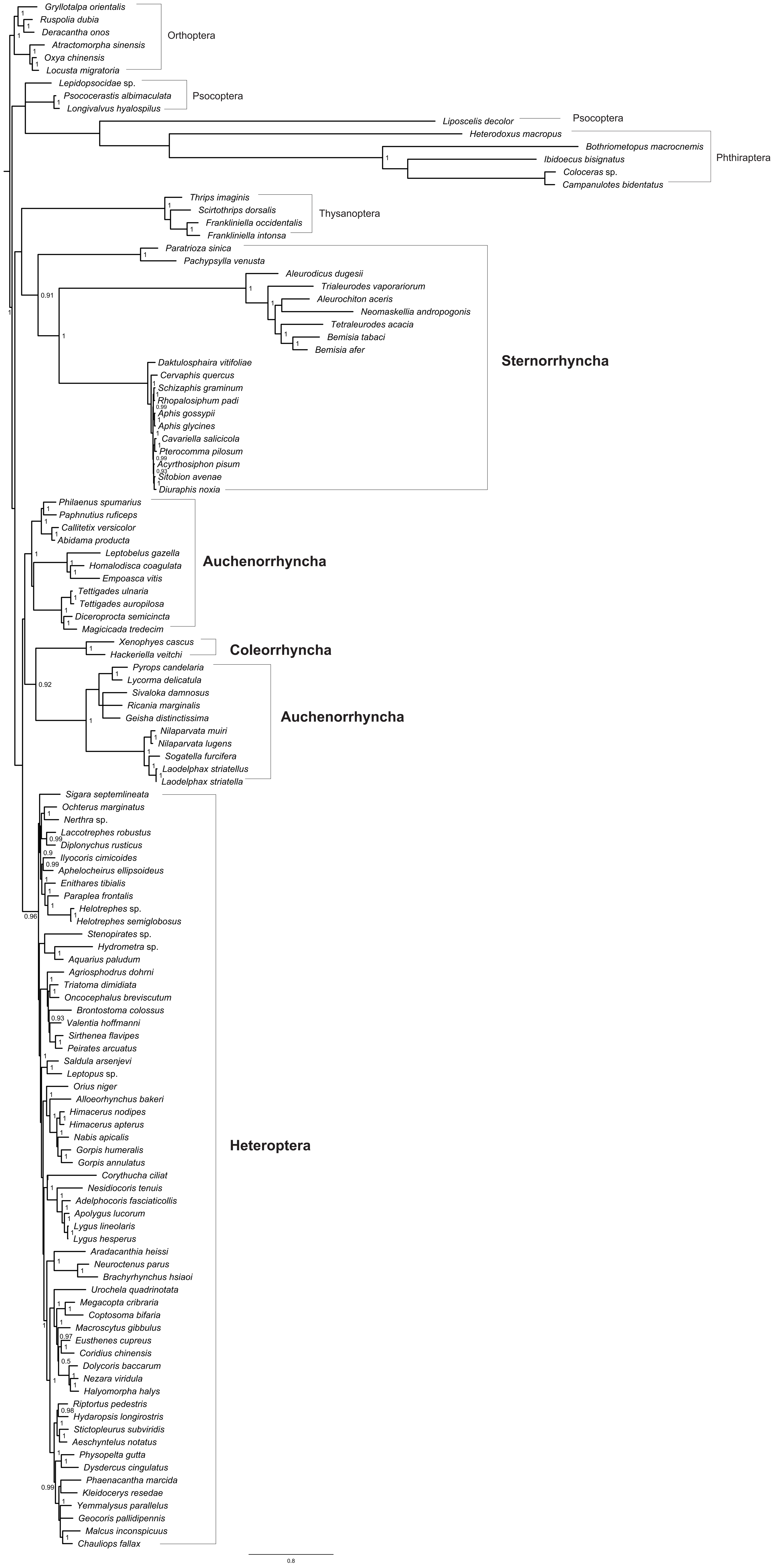

Fig. S2 Bayesian tree estimated from dataset of 122_taxa_PCGDegen under CATGTR model. Node numbers show posterior probabilities (above 0.9), and scale bar represents substitutions/site.
